# Students or medical professionals: whose knowledge improved after social-medicine training? Results from a quasi-experimental evaluation study

**DOI:** 10.1007/s00127-021-02197-4

**Published:** 2022-01-05

**Authors:** Beate Muschalla, Stefanie Baron, Theresa Klevers

**Affiliations:** 1 Technische Universität Braunschweig, Psychotherapy and Diagnostics, Humboldtstraße 33, 38106 Braunschweig, Germany; 2 Humboldt-Universität zu Berlin, Berlin, Germany; 3 Universität Potsdam, Potsdam, Germany

**Keywords:** ICF, Work ability, Mental health, Impairment, Rehabilitation professionals

## Abstract

**Purpose:**

Rehabilitation professionals are faced with judging and describing the social-medicine status of their patients. Rehabilitation professionals must know the core concepts of acute unfitness for work, psychological capacities, and long-term work capacity. Acquiring and applying this knowledge, requires training. The research question is if and to what extent medical professionals and students’ knowledge changes after social medicine training.

**Methods:**

This quasi-experimental study was carried out in the real-life context of social medicine training. Psychology students (*n* = 42), physicians/psychotherapists (i.e. state-licensed health professionals) (*n* = 44) and medical assistant professionals (*n* = 29) were trained. Their social medicine knowledge was measured before and after training by a 10-min expert-approved and content valid knowledge questionnaire. Three free-text questions had to be answered on the essential aspects of present and prognostic work ability and psychological capacities. Answers were rated for correctness by two experts. Paired *t* tests and variance analysis have been calculated for group comparisons.

**Results:**

All groups improved their social medicine knowledge from the pre- to the post-test. The students started with the lowest level of knowledge in the pre-test. After training, 69% of the physicians/psychotherapists and 56.8% of the medical assistant professionals, but only 7% of the students, obtained maximum scores for naming psychological capacities.

**Conclusions:**

Social medicine knowledge increased after a training course consisting of eight lessons. The increase was greater for medical assistant professionals and physicians/psychotherapists than for students. Social medicine training must be adjusted to the trainee groups’ knowledge levels.

## Introduction

Mental health problems are often associated with problems at work [1–4], poor working ability or even early retirement. Thereby it is not the symptoms themselves that explain the work (dis)ability, but capacity impairment [5–7]. Physicians, psychotherapists and other health professionals in (occupational) rehabilitation settings are faced with the task of judging and describing the social medicine status of their patients with mental health problems. One major question in social medicine is that of the patient’s work ability. To answer such social medicine questions, occupational health clinicians need to know the following:

- what “acute work ability/(un)fitness for work” means,
- which criteria have to be considered when judging “long-term work capacity/prognostic work ability”,
- in which “psychological capacity dimensions” can work ability be described.

Overall, clinicians need to consider not only the patient’s symptoms, but also their psychological capacity level. The diagnosis and description of psychological capacities is a relatively new field in clinical diagnostics, which until now has been unfamiliar to most clinicians. However, it is necessary for preparing precise social medicine reports and for choosing appropriate interventions.

This present research investigates if and to what extent, social medicine knowledge changes from before to after professional training, focusing on the above-mentioned contents. The contents of the training include work ability, psychological capacity dimensions, and the exploration and description of psychological capacity impairments, by means of the Mini ICF Rating for Impairment in Activities and Participation due to psychological disorders (Mini-ICF-APP), an internationally evaluated instrument for observer rating of mental capacity impairment.

The Mini-ICF-APP has become a standard instrument for social medicine descriptions of psychological capacities and work ability. The Mini-ICF-APP [8, 9] is an observer rating which has been internationally evaluated [10–13] and is established in social medicine practice. Social medicine guidelines suggest using it for work ability description and rehabilitation reports [14, 15]. The Mini-ICF-APP is used to quantify and differentiate capacity impairments in the context of mental disorders. It gives a selection of capacity dimensions, derived from the *International Classification of Functioning, Disability, and Health* (ICF) [16], which need to be assessed in social medicine judgments of work ability: (1) adherence to regulations, (2) planning and structuring of tasks, (3) flexibility, (4) competency, (5) competence to judge and decide, (6) endurance, (7) assertiveness, (8) contact with others, (9) group integration (10) intimate relationships, (11) non-work activities, (12) self-care, and (13) mobility. The anchor definitions for each item are provided in a rating manual [8]. However, the rating is complex, as raters must use all the available information, including the participant’s self-report, case records, and observations from the interview situation, and integrate this into their clinical judgment. Inter-rater reliability for the Mini-ICF-APP varies from *r* = 0.70 (untrained raters) to *r* = 0.90 (trained raters [8]).

For quality assurance purposes, i.e. to gain valid capacity ratings, health professionals involved in social medicine tasks must be trained. Successful training ensures that they are familiar with basic social medicine concepts and the psychological capacity dimensions.

In Germany, social medicine training, focusing on psychological capacity description with the Mini-ICF-APP, is regularly conducted with different groups. This training is conducted by the instrument’s developers [7, 8]. The aim of the training is to introduce the trainees to basic social medicine knowledge (i.e. criteria for acute unfitness for work/work ability, and for long-term work capacity/prognostic work ability), as well as the concept of psychological capacities and the capacity dimensions of the Mini-ICF-APP.

The importance of social medicine and training in social medicine competency has been stated throughout the world in recent years: (interdisciplinary) social medicine has become a part of the medical curriculum [17–24], but it is not yet regularly found in education for psychology or for other health professions. This may be due to the historical, curative traditions of many fields of health care, which mainly focus on symptoms and their reduction, i.e. acute treatment, instead of long-term management of chronic illness. Furthermore, medicine today is a broad field which requires specialization according to illness types, such as cardiology, neurology, orthopedics, and psychiatry. Although specialization in social medicine can also be done via a post-graduate certificate, this is only done by a few specialists. However, currently, social medicine knowledge is needed by much more than a few specialists because social medicine questions (such as prevention of work disability in mental disorders, or work adjustment for return to work) are becoming more important in many therapeutic fields. A prominent example is prevention and the treatment of people with work problems who take sick leave due to mental disorders, which is increasingly becoming a problem internationally [1, 2, 4, 6].

Until now, social medicine has seldom been taught in health professionals’ education. Evaluation studies on the outcome of social medicine training are also scarce [19, 25, 26]. Therefore, action and research in this important field are needed. As far as we know, this is the first evaluation of social medicine training focusing on psychological capacities and work ability in mental disorders. The training-addressed people who work or will work with mental health patients include the following: physicians and psychotherapists, occupational therapists, social workers, other medical assistant professionals, and undergraduate psychology students. The training’s evaluation therefore included three different groups: undergraduate psychology students, medical assistant professionals, and physicians or psychotherapists.

This research aims to investigate:

1. if the three groups students, physicians/psychotherapist, medical assistants – show higher social medicine knowledge in a test after the training, compared to a test before it and
2. if the development of social medicine knowledge from pre-test to post-test is different in the three trainee groups. Social medicine knowledge is operationalized by a knowledge test which is presented to the participants before and after the social medicine training. The test questions are shown in detail in Table 2.

## Materials and methods

This quasi-experimental study was carried out in the naturalistic (i.e. real life) context of social medicine training. Social medicine training is a course of eight lessons for students and health professionals who want to (or are required to) acquire knowledge in social medicine. The training (Table 1) includes concepts of acute unfitness for work and long-term work capacity, understanding of psychological capacities, and a description of work ability, based on the internationally established Mini-ICF-APP capacity dimensions [8, 10, 11].

**Table 1:** Social-medicine training’s structure and contents

Lesson (one lesson 50 min)	Contents	Training methods
1 + 2	Introduction Pretest social-medicine knowledge test Explanation of the social-medicine contents: Criterion for acute unfitness for work Judgment of chronic long term work capacity Introduction into the biopsychosocial health model of the ICF [16] Introduction into the capacity dimensions of the Mini-ICF-APP [7–9] Summary and questions to the lecture contents	Trainers introduce themselves (10 min) Participants fill in the knowledge test on their own (10 min) Lecture (25 min) Lecture (25 min) Group discussion (20 min)
3 + 4	Training phase with case vignettes: a case of agoraphobia and impaired mobility, a case of depression with impaired capacity for contacts with others Video demonstration: Exploration of a fictive patient concerning types and degrees of psychological capacity impairment. Exploration is done by a clinician who is social-medicine specialist	Applying the Mini-ICF-APP rating, group discussion and explanations by the trainers (50 min) Participants describe the degrees of capacity impairment according to Mini-ICF-APP, group discussion and explanations by the trainers (50 min)
5 + 6	Video demonstration: Exploration of a patient concerning types and degrees of psychological capacity impairment. Exploration is done by a clinicians who is social-medicine specialist	Participants describe the degrees of capacity impairment according to Mini-ICF-APP, group discussion and explanations by the trainers (100 min)
7 + 8	Demonstration of best practice examples of social-medicine reports with coherent descriptions of psychological capacities, and correct judgments on (un)fitness for work or long term work capacity Summary and questions concerning the case vignettes and video demonstrations Posttest social-medicine knowledge test Feedback and farewell	Lecture (60 min) and group discussion Questions and Answers (20 min) Participants fill in the knowledge test on their own (10 min) Trainers thank the participants, hand out material to read at home and offer contact for further discussion or questions on social-medicine issues and Mini-ICF-APP

### Procedure

In this investigation, the training was carried out with psychology students in one group and physicians/psychotherapists and medical assistant professionals (i.e. occupational therapists, social workers, and others) in other groups. Their social medicine knowledge was assessed using the same knowledge test, to be performed before and after the training. The students, physicians/psychotherapists, and medical assistant professionals were compared in terms of their knowledge development from before to after the training. The first knowledge test (pre-test) was given in the beginning of the training course, and the second (identical) knowledge test (post-test) was given in the end of the training course (Table 1). The time interval between pre- and post-test was about 7 h. Each training course comprises eight lessons of 50 min each, i.e. in sum a training course was about 7 h. All training courses were conducted on 1 day.

### Social medicine training with Mini-ICF-APP

Training in social medicine concepts and capacity description according to Mini-ICF-APP was performed with members of different professions: undergraduate psychology students (*n* = 42), medical assistant professionals (*n* = 44), and state-licensed physicians or psychotherapists (*n* = 29).

The training was given by two of the authors (BM, SB) in typical continuing education settings, i.e. in an education academy of the German federal pension agency, in an institute for psychotherapy education, and in classrooms at two universities. The trainers are both state-licensed psychotherapists who have had continuing education in psychosomatic rehabilitation and social medicine. They are experts in social medicine judgment and psychological capacity description, and are two of the developers of the Mini-ICF-APP [7, 8]. Both have more than 10 years of professional experience. They perform social medicine diagnostics and give rehabilitation treatment, as well as social medicine training. They are established experts in their field, have given more than 100 social medicine training courses and are listed as experts for social medicine and social law by the Berlin psychotherapist’s professional association.

The training’s contents, structure and methods are set out in detail in Table 1. All the training courses have been conducted according to this scheme. Course contents were new for all participants.

### Participants

We used a convenience sample, i.e. locations where these social medicine trainings are routinely conducted with students and health professionals as participants. Training courses which were given by the trainers BM and SB in the year 2017 were included. The training was conducted at two universities (with 42 undergraduate psychology students) and in two institutes for continuing professional education (with 44 medical assistants and 29 psychotherapists/physicians). All 115 subjects participated voluntarily in the pre- and post-tests and gave informed consent.

The physicians/psychotherapists and medical assistants attended the training voluntarily, i.e. they were driven by personal interest. They work in inpatient medical rehabilitation or in outpatient settings. They are all faced with social medicine questions in their working routine.

*Physicians and psychotherapists* constitute a group due to their similar qualification path and level as state licensed health professionals. In contrast to medical assistants or students, they are allowed to diagnose patients and provide treatment on their own; they are not bound by other people’s instructions.

*Medical assistant personnel* can be described as co-therapists who are involved not primarily in symptom therapy (such as the physicians) but in capacity observation, capacity description, and capacity training therapies of different types. “Capacity-orientation” is this the common characteristic of the medical assistants, even if they have different basic professions and target different life domains (workplace, mobility, general life). There were 3 occupational therapists (ergotherapists), 26 psychologists (without psychotherapy qualification), 11 social workers, 2 nurses, 2 others.

For the *psychology students*, the social medicine training was part of their curriculum. Students participated in the training course during the first part of their academic education, i.e. in the middle (third semester) of their undergraduate psychology studies. Undergraduate health psychology students were included in this study because they are the next generation of psychologists who will do work ability- and capacity-description later in their professional life, e.g. in hospitals, rehabilitation settings etc. Psychologists are educated to become experts in capacity description and diagnostics of psychological capacities. Therefore, they must be considered as a relevant group who should learn about work ability and capacity exploration and -description in their education.

### Social medicine knowledge test

The questions and tasks in the social medicine knowledge test are shown in detail in Table 2. They include three main knowledge contents which form the core content of the training. The contents of the test questions are based on standard social medicine literature used in continuing education [8, 14, 33]. Thus, they represent the current gold standard of expertise in this field. The knowledge test is content valid, which has been checked by expert’s revisions in an earlier pilot phase before the study. The three test questions and corresponding correct answers have been formulated by one author of the Mini-ICF-APP working group, and have then been reviewed and approved by coauthors who are all year-long experienced social-medicine experts in research and clinical practice.

**Table 2:** Ratings of free answers in the knowledge test by rater A and rater B

	Question (Correct answer) [maximum knowledge score]	Rater A	Rater B	*T* Test *p*	Spearman correlation rater 1 and rater 2
Pretest	Please name four psychological capacities (Capacities according to Mini-ICF-APP are named and no psychopathology items. Capacities are, e.g. capacity for structuring and planning, flexibility, endurance, judgment, adjustment, etc.) [4]	1.30 (1.29)	1.47 (1.21)	0.003	0.873**
	Please name the required criteria for judgment of chronic long term work capacity (history of illness and course of capacity impairment, treatment history and treatment options, prognosis of illness and capacity status) [1]	0.06 (0.24)	0.07 (0.27)	0.319	0.931**
	Criterion for acute unfitness for work (Need for help in conducting the individual work tasks, the reason for unfitness is due to illness-impairment which affects relevant capacities) [1]	0.00 (0.00)	0.03 (0.18)	0.045	–
Posttest	Please name four psychological capacities [4]	2.66 (1.52)	2.73 (1.50)	0.070	0.946**
	Criteria for judgment of chronic long term work capacity [1]	0.22 (0.42)	0.22 (0.42)	–	1.000**
	Criterion for acute unfitness for work [1]	0.10 (0.31)	0.24 (0.439	0.000	0.616**

*T* test for paired samples. *N* = 115

^**^p < 0.001

Filling in the knowledge test takes 10 min. Objectivity was given due to a standardized written instruction and questions and a standardized evaluation of the correctness of the test answers by two raters. A solution manual was available for the raters who had to rate the correctness of the participants´ answers to the test questions. In the solution manual, the expected correct answers were listed. The way of constructing and using the knowledge test is similar to what is typically done in present research on knowledge evaluation [e.g. 27, 28].

The knowledge test contained three tasks requiring free answers. An author (BM, rater B) and a social medicine trained study assistant (TK, rater A) rated the correctness of the training participants’ answers. A manual with examples of correct answers for each question had been prepared in advance. The raters had to give knowledge scores for each answer, according to its degree of correctness (Table 2). The inter-rater reliability between BM and TK was between *r* = 0.616** and *r* = 1.000 (Table 2) and was therefore satisfactory. Regarding the level of knowledge scores, rater A was slightly stricter than rater B, as the scores she gave for the participants’ answers were consistently lower than those given by rater B.

The first question in the knowledge test asked the participants to name “psychological capacities”. This question could be answered by naming four different capacity dimensions, according to the Mini-ICF-APP. Correct answers were those which named capacities in the sense of categories of humans’ activities and action potentials (e.g. what people can do), and explicitly not psychopathology (i.e. symptoms that people may suffer from). One point was given for each correctly named psychological capacity. The maximum possible score for this question was four points.

The second question pertained to the concept of work ability. Participants were asked to name the criterion for an “acute unfitness for work” in the case of a mental illness. The correct answer was acute unfitness for work is justified when an illness-based capacity impairment occurs to the extent that support from third parties is required. This means that colleagues must help and take on some tasks, but this help cannot be provided regularly.

One point was given for the correct answer.

The third question asked for the additional criteria necessary for reaching a conclusion about prognostic work ability, i.e. “long-term work capacity”. The correct answer is in addition to the present (acute) capacity status, the course of the illness and the capacity status must also be considered. Integrating the information about the illness’ course is a requirement for making a prognostic statement. The course includes the history of the mental health condition’s symptoms and capacity impairments, previous attempted treatments, possible remaining treatment options, and conclusions about whether the illness-based capacity impairments might change (e.g. capacity status improves or decreases) or remain stable. One point was given for the correct answer.

### Data analysis

The reliability of the judgments about correct answers has been checked as a basic technical requirement of the study. This was done by calculating the inter-rater-reliability of the two raters A and B, who checked the participants’ answers in the knowledge tests (Table 2). For further descriptive purposes, the inter-relations between the knowledge score in the pre- and post-tests are reported in Table 3.

**Table 3:** Spearman correlations between knowledge scores in tests pre and post training

	Pre-psychological capacities	Pre-long-term work capacity	Pre-acute unfitness for work	Post-psychological capacities	Post-long-term work capacity
Pre-long-term work capacity	0.088				
Pre-acute unfitness for work	− 0.037	0.321**			
Post-psychological capacities	0.555**	− 0.006	− 0.096		
Post-long-term work capacity	0.297**	0.269**	0.014	0.267**	
Post-acute unfitness for work	0.170	0.090	0.007	0.192	0.303**

Level of significance: **p<.01, *p<.05

The research question was explored using multivariate analysis of variance with repeated measurement, including Bonferroni correction (Table 4). The effects of repeated measurement and the interaction effect of repeated measurement and group (students, medical assistant, physicians/psychotherapist) were calculated to explore the development of knowledge from pre- to post-test, and to explore potential group differences in this development.

**Table 4:** Development of social-medicine knowledge pre post training: comparison of students, medical assistant professionals and physicians/psychotherapists. *N* = 115

	Pretraining *M* (SD) [% maximum score]	Post-training *M* (SD) [% maximum score]	Multivariate tests Pillai’s trace repeated measurement	Interaction effect repeated measurement × group
Psychological capacities				
Students (*n* = 42)	0.76 (0.53) [0%]	1.21 (1.46) [7.1%]	0.308 *F*(1,112) = 49.94 *p* = 0.000 *ε*^2^ = 0.308	0.060 *F*(2,112) = 3.54 *p* = 0.032 *ε*^2^ = 0.060
Medical assistant professionals (*n* = 44)	1.93 (1.44) [20.5%]	3.05 (1.31) [56.8%]		
Physicians/psychotherapists (*n* = 29)	1.79 (1.08) [3.4%]	3.03 (1.59) [69%]		
Acute unfitness for work				
Students (*n* = 42)	0.05 (0.22) [4.8%]	0.07 (0.26) [7.1%]	0.180 *F*(1,112) = 24.53 *p* = 0.000 *ε*^2^ = 0.180	0.084 *F*(2,112) = 5.16 *p* = 0.007 *ε*^2^ = 0.084
Medical assistant professionals (*n* = 44)	0.02 (0.15) [2.3%]	0.32 (0.47) [31.8%]		
Physicians/psychotherapists (*n* = 29)	0.03 (0.19) [3.4%]	0.35 (0.48) [34.5%]		
Long-term work capacity				
Students (*n* = 42)	1.01.011.0.0 (0.00) [0%]	0.047 (0.22) [4.8%]	0.108 *F*(1,111) = 13.4 *p* = 0.000 *ε*^2^ = 0.108	0.043 *F*(2,111) = 3.49 *p* = 0.087 *ε*^2^ = 0.043
Medical assistant professionals (*n* = 44)	0.05 (0.21) [2.3%]	0.30 (0.46) [31.8%]		
Physicians/psychotherapists (*n* = 28)	0.21 (0.42) [20.7%]	0.36 (0.49) [35.7%]		

## Results

### Technical and descriptive data

The three participant groups (students, physicians/psychotherapists, and medical assistants) began the training with different basic knowledge. On average, the physicians/psychotherapists and medical assistants have been working with people with mental health problems for *M* = 8.4 (SD = 7.0) and *M* = 8.8 (SD = 7.5) years. These professionals are on average *M* = 42.8 (SD = 15.3) years of age. The students have not yet begun working with patients and were younger (*M* = 25.1 years, SD = 7.8, *p* < 0.001).

Table 2 illustrates the inter-correlation between the two raters A and B, who checked the correctness of the answers in the knowledge test.

Table 3 shows the inter-correlations of the knowledge scores. The knowledge scores for psychological capacities were most consistently positively related pre-post (*p* = 0.555**), whereas the knowledge scores for acute unfitness for work did not correlate pre-post (*p* = 0.007).

### Research question: social medicine knowledge pre and post training

Table 4 shows the group comparison of knowledge scores before and after the social medicine training. All the groups improved in knowledge from pre- to post-test (repeated measurements *p* = 0.000, Table 4). However, differences can be seen between the groups: of all the groups, students started from the lowest level of knowledge in the pre-test. Medical assistant professionals already had higher scores in the pre-test for the task naming psychological capacities: one-fifth of the medical assistant professionals (20.5%) named capacities correctly, but only 3.4% of the physicians/psychotherapists and 0% of the students. In contrast, the physicians/psychotherapists had higher pre-test knowledge of the criterion for long-term work capacity description: 20.7% name the criterion correctly, in contrast to only 2.3% of the medical assistant professionals and 0% of students.

After training, 69% of the physicians/psychotherapists and 56.8% of the medical assistant professionals but only 7% of the students attained the maximum score for naming capacities. Thus, the steepest learning rate was seen in the group of physicians/psychotherapists. A similar pattern of learning development can be seen for the other knowledge questions, i.e. the criteria for unfitness for work and long-term work capacity. In contrast to the first question (naming capacities), only one-third of the physicians/psychotherapists and medical assistant professionals achieved maximum scores in the post-test, and less than 8% of the students did so. The scores for the development of knowledge are significantly different among the three groups (interaction effects *p* = 0.032, *p* = 0.007, *p* = 0.087, Table 4).

## Discussion

All three groups—students, physicians/psychotherapists, medical assistant professionals—showed improvement in their social medicine knowledge. However, different patterns of improvement from pre- to post-training can be observed in the three groups. This data provides some points for discussion:

Why do *medical assistant professionals* start with a higher pre-test knowledge of psychological capacities than physicians/psychotherapists? A possible explanation might be that medical assistant professionals are, from their education and their role in the rehabilitation team, trained in describing present observable activities, behavior, and capacity [14]. They may even be more familiar with this level of description and diagnosis than physicians and psychotherapists. *Physicians/psychotherapists*, on the other hand, learn to diagnose and differentiate illness symptoms rather than capacities. They also learn differential diagnosis, which requires knowledge of illness development and treatment options during the course of illness. This knowledge of illness development may, however, explain why physicians/psychotherapists start with a higher pre-test knowledge of the criterion for long-term work capacity.

All groups increased their knowledge about the three selected social medicine core components (capacities, criteria for unfitness for work, long-term work capacity), but by very different amounts. Whereas physicians/psychotherapists achieved the highest knowledge scores in the post-test, and medical assistant professionals achieved a moderate improvement, the student sample only achieved a small increase. A reason for these different amounts of improvement may be the different starting levels, i.e. *students* do not have any experience in clinical practice yet and are not as familiar with clinical diagnosis as physicians, psychotherapists, and medical assistant professionals. Basic clinical experiential knowledge (in the sense of crystallized intelligence [29]) seems to be necessary for learning social medicine. Psychopathology and anamnesis are essential basic concepts upon which the additional social medicine competencies (exploration, description and judgment of work ability and capacity impairment) are built [9, 14]. The theoretical knowledge, e.g. on the nature and types of mental disorders, which is taught in undergraduate psychology courses may be not sufficient to allow for the immediate addition of social medicine training. Furthermore, the fact that the training course was mandatory for the students may have brought about differences in motivation to participate and may also be a rationale for the lower increase of knowledge in the students.

We investigated a convenient sample in a real-life context, and did not exclude persons. We investigated “typical” persons who normally participate in such trainings in real life.

We focused on social medicine basic knowledge, according to the usual social medicine guidelines [8, 9, 14, 15]. The present investigation is the first to examine the improvement in basic social medicine knowledge.

This present research is timely and has high practical relevance for three reasons: first, social medicine knowledge is a core competence needed by rehabilitation professionals, because rehabilitation means the treatment of chronic illness. Chronic mental illness is a high load worldwide. Chronic illness management needs social medicine interventions and diagnostics. Second, the Mini-ICF-APP (which is a core topic in social medicine training) is an internationally evaluated and practically applied instrument for the assessment of work ability and psychological capacity status [10–13]. Third, to prevent work disability and find the right (work) support aids, writing precise and comprehensive reports on patient’s impairments is a core duty of (occupational) health professionals. This is also a task for preventive occupational medicine.

## Limitations and further research

This is a quasi-experimental study which was carried out in naturalistic training environments, i.e. in routine continuing education for health professionals, and within the mandatory curriculum for a bachelor of psychology (in courses on diagnosis and rehabilitation). Therefore, external validity can be assumed. The training investigated here is a basic course: the participants do need further education and clinical training to train their social medicine abilities. However, such short trainings with the aim of knowledge increase are not unusual [27].

We did not investigate a control group. Thus, we do not have a group for comparison who did not receive the training. This may endanger internal validity.

We focused on social medicine basic knowledge, according to the usual social medicine guidelines [8, 9, 14, 15]; this knowledge was assessed in free reproduction mode. Different results might have occurred if other test formats (e.g. multiple-choice questions) had been used, or other content had been chosen (e.g. therapeutic content).

There was no follow-up; therefore, we cannot say if the social medicine knowledge acquired remained stable over a longer period of time. However, an initial increase in knowledge is a requirement for improvement in social medicine diagnostics. To give a valid decision on (un)fitness for work for a specific patient requires that the health professional is aware of the criteria for unfitness for work.

To investigate whether or not social medicine knowledge is being used adequately in clinical practice, further research is needed on the quality of social medicine decisions in rehabilitation practice and the reasons for these decisions. In the German rehabilitation context, a quality check of social medicine reports by peer raters has been initiated. In this, clinicians’ work ability decisions and descriptions of capacities are particularly checked for plausibility by the peer raters [20, 30, 31]. Training for clinicians (including the training investigated here) may be useful for further optimizing the quality of social medicine reports.

## Conclusion

Social medicine knowledge increased, in comparison to a pre-test, after a social medicine training course consisting of eight lessons. The increase in knowledge was greater in medical assistant professionals and physicians/psychotherapists than in undergraduate psychology students.

In future, the training methods, content, and duration might be adjusted to suit specific groups. Students with theoretical knowledge but no clinical experience might need some clinical training in practice, e.g. through internships or exercises, before learning social medicine. Practical experience of clinical basics could pave the way for better understanding of complex social medicine content: in the practice settings, students should learn that clinical diagnosis is not simply checking symptoms against a list; it also requires perception about the interactions between symptoms, psychological capacities, and context factors. An understanding of these interactions is essential for social medicine, e.g. for questions of work performance and work ability [32, 33].

If social medicine is taught to beginners prior to clinical experience, it should be done with methods that directly establish practical relevance, such as example videos (as have been used in our training), case concepts, role-plays, to illustrate clinical judgment practically even at entry level.

## Data Availability

The datasets used and/or analyzed during the current study are available from the corresponding author on request.

## Abbreviation

ICF: International Classification of Functioning, Disability and Health
